# Developing Biliary Atresia-like Model by Treating Human Liver Organoids with Polyinosinic:Polycytidylic Acid (Poly (I:C))

**DOI:** 10.3390/cimb44020045

**Published:** 2022-01-27

**Authors:** Patrick Ho-Yu Chung, Rosana Ottakandathil Babu, Zhongluan Wu, Kenneth Kak-Yuen Wong, Paul Kwong-Hang Tam, Vincent Chi-Hang Lui

**Affiliations:** 1 Department of Surgery, Li Ka Shing Faculty of Medicine, The University of Hong Kong, Hong Kong, China; chungphy@hku.hk (P.H.-Y.C.); hannawu@hku.hk (Z.W.); kkywong@hku.hk (K.K.-Y.W.); paultam@hku.hk (P.K.-H.T.); 2 MRC WIMM Centre for Computational Biology, Medical Research Council, Weatherall Institute of Molecular Medicine (WIMM), John Radcliffe Hospital, University of Oxford, Oxford OX3 9DU, UK; rosana.ottakandathilbabu@ndm.ox.ac.uk

**Keywords:** biliary atresia, liver, inflammation, organoids, transcriptomic, Polyinosinic:Polycytidylic acid

## Abstract

Background: We explored the feasibility of creating BA-like organoids by treating human liver organoids with Polyinosinic:Polycytidylic acid (Poly I:C). Methods: Organoids were developed from the liver parenchyma collected during Kasai portoenterostomy (BA) and surgery for other liver disorders (non-BA). The non-BA organoids were co-cultured with poly I:C (40 µg/mL). The organoid morphology from both samples was compared on day 17. RNA-sequencing was performed to examine the transcriptomic differences. Results: Non-BA liver organoids developed into well-expanded spherical organoids with a single-cell layer of epithelial cells and a single vacuole inside. After poly I:C treatment, the majority of these organoids developed into an aberrant morphology with a high index of similarity to BA organoids which are multi-vacuoled and/or unexpanded. RNA-sequencing analysis revealed that 19 inflammatory genes were commonly expressed in both groups. Conditional cluster analysis revealed several genes (SOCS6, SOCS6.1, ARAF, CAMK2G, GNA1C, ITGA2, PRKACA, PTEN) that are involved in immune-mediated signaling pathway had a distinct pattern of expression in the poly I:C treated organoids. This resembled the expression pattern in BA organoids (*p* < 0.05). Conclusions: Poly I:C treated human liver organoids exhibit morphology and genetic signature highly compatible to organoids developed from BA liver samples. They are potential research materials to study immune-mediated inflammation in BA.

## 1. Introduction

Biliary atresia (BA) is a devastating congenital anomaly characterized by inflammatory fibrosclerosing changes of the extra- and intra-hepatic biliary systems. It is widely believed that most BA start with a series of abnormal immune interactions that trigger biliary inflammation as the common pathway. The critical role of inflammation in the pathogenesis of BA has been well established [1]. As a result of the biliary inflammation, the bile duct undergo irreversible fibrotic changes and bile flow is obstructed. Even though it is the most frequent cause of persistent neonatal cholestasis, its actual incidence remains low globally. Asia-Pacific countries have reported the highest incidence, which is around 1.3 to 2.0 new cases per 10,000 live births per year. The incidence is even lower among Caucasians, where there are only around 0.5 to 0.8 new cases per 10,000 live births per year [2].

Clinically, affected patients present with obstructive jaundice that persists beyond the neonatal period. Liver failure will follow if the disease is allowed to progress without the appropriate treatment. Kasai portoenterostomy (KPE), which consists of the excision of the fibrotic bile duct, and portoenterostomy is the most widely accepted corrective surgery for BA but the treatment outcome remains suboptimal. After KPE, only around half of the patients can achieve long term survival without liver transplant [3]. Native-liver survivors are prone to develop life-long morbidities, including portal hypertension, recurrent cholangitis, and growth impairment [4].

Over the years, there has been an active research on new therapies that could enhance the curative rate of BA. In recent years, the introduction of organoid technology has opened up a new avenue for research in hepatobiliary diseases. Previous reports have confirmed that BA liver organoid could be successfully developed from human samples and used as scientific research models [5]. Unfortunately, BA research has been limited by its low incidence and only a scant amount of clinical samples are available for experimental purposes. Not uncommonly, the samples collected during KPE represent the late stage of the disease when liver parenchyma is replaced by fibrotic tissues. The early inflammatory process, which is potentially reversible, cannot be captured. BA animal models have been developed as the study tools but they are not human-based [6]. As BA is widely believed to start with a viral induced inflammation [7], we aimed to replicate the inflammatory process in BA and create a disease-like model by co-culturing liver organoids with immunogenic components of the virus. Moreover, the morphology and the genetic profile of this organoid model will be compared with those BA samples collected from patients to determine the similarity. To our best knowledge, this is the first report of this research model in the literature.

## 2. Materials and Methods

### 2.1. Samples

All the liver samples were collected from non-BA and BA patients attending the Department of Surgery, Queen Mary Hospital, Hong Kong. The diagnosis of non-BA control samples were: (i) hepatoblastoma (HB); (ii) choledochal cyst (CC); and (iii) cholestatic disease (CS) (Table 1). The non-tumor part of hepatoblastoma, biopsy specimens from CC and CS were used to generate liver organoids as non-BA control samples. In this study, the diagnosis of BA was confirmed with surgical exploration, revealing an atretic bile duct and histological analysis. All of them suffered from isolated BA. Liver biopsy from BA was performed by wedge resection during the KPE and each sample measured 0.5 cm × 0.5 cm in size. This study has been approved by the institutional ethics committee (IRB no: UW 20-723) and was performed according to a standard guideline set up by the competent authority.

### 2.2. Growing of Liver Organoids

The generation of organoids from liver biopsy was performed according to our previously described protocol [8]. Briefly, the liver biopsy was minced and digested in gentleMACS-C Tube (Miltenyi Biotec Inc., San Jose, CA, USA) with 5 mL digestion medium (Multi Tissue Dissociation Kit 1; Miltenyi Biotec Inc., San Jose, CA, USA), filtered (70 µm, 30 µm) and magnetic sorted using human CD326 (EpCAM; Miltenyi Biotec Inc., CA, USA) magnetic beads. The obtained CD326 positive cells were mixed with Matrigel (50,000 cells in 50 µL; 356231; Corning Biocoat, Corning, NY, USA). After solidification of the Matrigel at 37 °C for 5 min, organoid medium (Advanced DMEM/F12 supplemented with 1% Penicillin/Streptomycin (Invitrogen, Carlsbad, CA, USA), 250 ng/mL Amphotericin B (GIBCO, Waltham, MA, USA), 1% N2 and 1% B27(GIBCO), 1.25 mM N-Acetylcysteine (Sigma, Burlington, MA, USA), 10 nM gastrin (Sigma, Burlington, MA, USA), and the growth factors: 50 ng/mL mEGF (Peprotech, East Windsor, NJ, USA), 100 ng/mL FGF10 (Peprotech, East Windsor, NJ, USA), 25 ng/mL HGF (Peprotech, East Windsor, NJ, USA), 10 mM Nicotinamide (Sigma, Burlington, MA, USA), 5 µM A83.01 (Tocris, Bristol, UK), and 10 µM FSK (Tocris, Bristol, UK). Then, 25 ng/mL Noggin (Peprotech), 500 ng/mL R-Spondin 1 (R&D), 100 ng/mL Wnt3a (R&D), 10 µM Y27632 (Sigma Aldrich, Burlington, MA, USA)) was added to the culture. Cultures were incubated at 37 °C and medium was changed once every two days.

### 2.3. Treatment of Non-BA Control Liver Organoids with Poly I:C

To prepare single cells from control liver organoids, the organoid medium, along with matrix gel containing organoids, was aspirated from each well, transferred to a 15 mL tube, 1–2 mL cold Advanced DMEM/F12 was added, and the mixture was incubated on ice for 10 min to dissolve matrix gel before centrifugation (300× *g*; 5 min). The supernatant was aspirated until the organoid pellet and a layer of matrix gel remained. Then, 1 mL 5X TrypLE Express (12604013; GIBCO, Waltham, MA, USA) was added, mixed well and incubated at 37 °C for 5 min. Then, 1 mL of cold Advanced DMEM/F12 was added to the mixture, which was pipetted up and down for 40–50 times with a small circumference opening glass pipette (diameter 0.3–0.5 mm) to efficiently dissociate organoids into single cells. Then, 5 mL cold Advanced DMEM/F12 medium was added, and the suspension was passed through a 30 µm strainer before centrifugation (300× *g*; 5 min) at 4 °C. The supernatant was aspirated until only the pellet remained and the cells were counted following addition of 200 µL Advanced DMEM/F12 with 10% FBS. The cells were then counted using Invitrogen Countess II FL Automated Cell Counter. Same number of organoid cells (1000) were mixed with 50 µL of Matrigel (Corning Biocoat), and added to each well of a 4-well plate (14444, Thermo Scientific, Nunc, Waltham, MA, USA). After solidification of the Matrigel at 37 °C for 5 min, 0.5 mL of organoid medium was added to each well and incubated at 37 °C. Poly I:C was added to the organoid cultures at day 3 (at final concentrations of 100 µg/mL, 40 µg/mL or 1 µg/mL) and cultured for 14 days. Two replicates were maintained for each treatment and experiments were performed on liver tissue-derived organoids of two HB patients and 1 CC patient.

### 2.4. Counting of Organoids

To quantify organoids with different morphologies, the number of organoids of well-expanded mono-cystic, multi-vacuoles, or un-expanded structures in each well was counted. The percentages of organoids of different morphologies were calculated by dividing the number of organoids of a particular morphology by the total number of organoids in each well. The percentages were expressed as mean ± SD.

### 2.5. RNA-Sequencing Analysis

Organoids were retrieved from Matrigel to individual tubes (one organoid per tube) for RNA sequencing. Organoid lysis, total RNA preparation, reverse transcription, amplification, and library construction were performed using single cell RNA-seq technology (Smart-seq2.0) with minor modifications for generating bulk RNA transcriptomics to obtain good depth in the transcriptome analysis [8]. The quality of the pre-amplified products of normal and BA organoids was confirmed by Bioanalyzer. Library construction was performed using Nextera XT Kit following the manufacturer’s protocol. Libraries were pooled and sequenced by pair ends of 100 base pairs (PE100) on an illumine HiSeq 2500 System.

### 2.6. Transcriptome Analysis

The bulk RNA-seq reads were subjected to quality check and bioinformatics analysis following our previously published analysis pipeline for the identification of differentially expressed (DE) genes between different groups [9]. Quality check was first performed using FastQC version 0.11.8 [10]. Adapter contamination and low-quality regions were further filtered using Cutadapt version 1.16 with the parameter −q = 33, and only reads with length ≥ 30 were retained [11]. Subsequent filtering for rRNA sequence contamination by alignment to human rRNA sequences using Bowtie version 2.3.4.3 with default parameters was performed [12]. Reads were mapped to the human reference genome (GRCh38). The transcriptome mapping/alignment and identification of exon–exon splice junctions with the human genome reference were performed using TopHat version 2.1.1 with default parameters [13]. All the samples had an overall alignment of >80% with human reference. HTSeq version 0.11.1 was used for counting of aligned reads per gene for differential expression analysis [14]. Normalization of gene expression count data and identification of DE genes were completed using R Limma-voom algorithm and DESeq2 version 1.18 [15,16]. Cut-off criteria to filter out low expression genes were CPM threshold value of 1 or log-CPM value of 0 using Limma-voom. DE genes were taken with an adjusted *p*-value cut-off of 0.05 for all analysis. The Gene Ontology analysis was performed for each cluster of genes by using the Gene Ontology database, the David Web Tools and PANTHER (http://www.pantherdb.org/), (accessed on 15 November 2021). R/Bioconductor packages was used for visualization [17,18,19]

## 3. Results

### 3.1. Poly I:C Treated Liver Organoids Were Morphologically Similar to BA Liver Organoids

**Figure 1:**
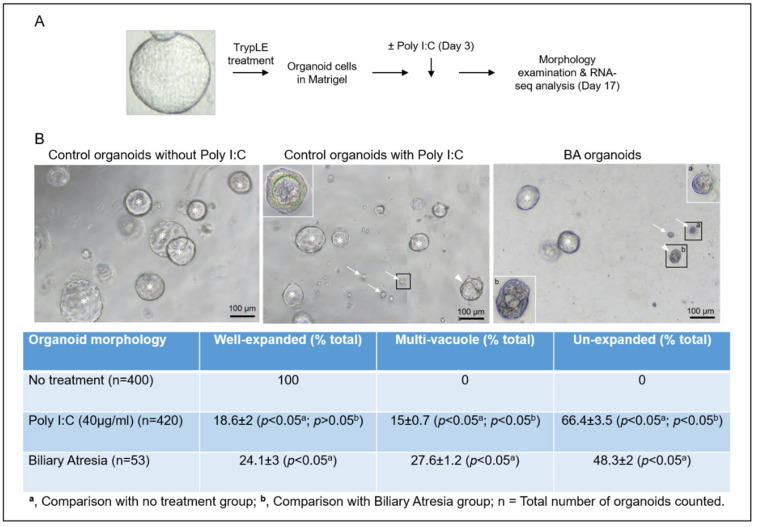
Poly I:C treated liver organoids were morphologically similar to BA liver organoids. (**A**) Schematic diagram of poly I:C treatment of normal liver tissue-derived organoids. (**B**) The morphology of liver organoids on day 17 from control samples without and with poly I:C treatment, and BA liver tissue-derived organoids (*, well-expanded mono-cystic spherical organoids with a single-cell layer of epithelial cells; arrowheads, poorly expanded structures with multiple vacuoles; arrow, tiny unexpanded cell clusters). The percentages (mean ± SD) of organoids of different morphologies in different groups were quantified and tabulated as shown.

To investigate if poly I:C influenced the organoid growth, we added poly I:C to the day 3 control liver organoids (HB and CC) and cultured for 14 days (Figure 1A). A total of 126 liver organoids were developed (non-BA control = 46; BA = 30; poly I:C treated control = 50). Without poly I:C, non-BA control organoids developed well-expanded cystic spherical shapes with a single outer layer of epithelial cells and a single vacuole inside (asterisk, Figure 1B). No organoids were formed in the culture with 100 µg/mL of poly I:C (Data not shown). In the culture with 40 µg/mL Poly I:C, organoids were developed into a well-expanded cystic structures (asterisk; well-expanded), poorly expanded structures with multiple vacuoles (arrowheads; multi-vacuole) or tiny unexpanded cell clusters (arrows; un-expanded) (Figure 1B). In the culture with 1 µg/mL, only well-expanded single vacuolated cystic spherical organoids with a single outer layer of epithelial cells were formed (Data not shown). In BA liver tissue-derived organoids culture, well-expanded single vacuolated cystic spherical organoids, poorly expanded structures with multiple vacuoles (arrowheads) or tiny unexpanded cell clusters were developed (Figure 1B). The percentages of well-expanded single vacuolated cystic organoids, poorly expanded structures with multiple vacuolated organoids, or tiny unexpanded organoids in control, poly I:C treatment, and BA groups were determined.

Morphologically, all control liver organoids developed into well-expanded spherical organoids with a single-cell layer of epithelial cells and a single vacuole inside. In contrast, after poly I:C co-culturing, the majority of these organoids developed into an aberrant morphology comparable to those organoids from BA livers (Figure 1B; multi-vacuole (15 ± 0.7%) or un-expanded (66.4 ± 3.5%) in poly I:C group vs. multi-vacuole (27.6 ± 1.2%) or un-expanded (48.3 ± 2%) in the BA liver).

### 3.2. Poly I:C Treated Organoids and BA Organoids Displayed Similar Transcriptomic Signatures

**Figure 2:**
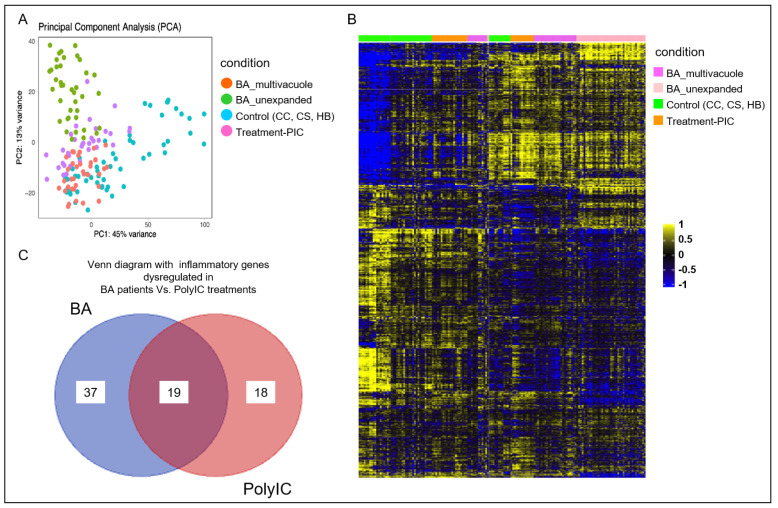
Poly I:C treated organoids and BA organoids displayed similar transcriptomic signatures. (**A**) Principal Component Analysis (PCA) showing clustering of n = 156 bulk transcriptomes from organoid samples from poly I:C treatment, controls (CC, SC, HB) and BA, each dot represents an organoid. (**B**) Heatmap showing the mRNA expression levels of total genes (1356 nos.) that are differentially expressed poly I:C treatment when compared to controls (yellow-high expression; blue—low/no expression), and BA. Each column represents a gene and each row represents an organoid from the conditions indicated by color bars to the right. (**C**) Venn diagram showing inflammatory genes dysregulated in BA patients versus poly I:C treated control organoids.

**Figure 3:**
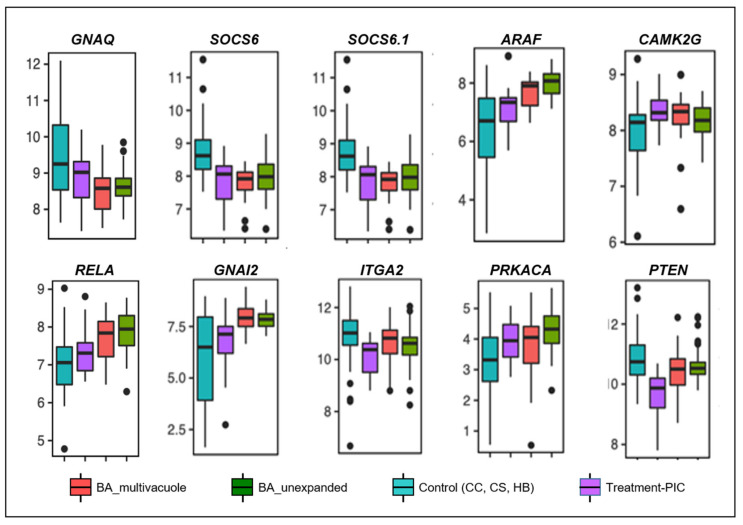
Boxplot showing the expression patterns of several genes that are involved in immune-mediated chemokines/cytokines signaling pathway and cellular metabolism that are differentially regulated in poly I:C treatment and BA at *p* < 0.05 (two-tailed Student’s *t*-test).

To further analyze if poly I:C treated organoids were similar to BA liver tissue-derived organoids, 35 poly I:C treated organoids were subjected to RNA-seq analysis to generate the bulk transcriptomics of each poly I:C treated organoids. Bioinformatics analysis of poly I:C treated organoids with our recent published transcriptomic data of non-BA control and BA organoids was performed (9). Principal Component Analysis (PCA) (Figure 2A) revealed that the transcriptomic signature of poly I:C treated organoids (purple dots) was distinct from the non-BA control liver tissue-derived organoids (blue dots). PCA also revealed that revealed a clustering of bulk transcriptomes from poly I:C treated control organoids (purple dots) that are close to BA organoids (green and orange dots) when compared with the untreated control organoid (blue dots). Analysis of mRNA expression levels profile of total 1356 genes revealed similar results obtained from poly I:C treated organoids and BA organoid when compared to other non-BA controls (Figure 3). Among the 56 and 37 inflammatory genes being differentially expressed in the BA and poly I:C treated samples, there were 19 genes that are common in both groups (Figure 2C: Venn diagram). They were selected for conditional cluster analysis and several genes that are involved in immune-mediated chemokines/cytokines signaling pathway and cellular metabolism (RELA, SOCS6, SOCS6.1, ARAF, CAMK2G, GNA12, ITGA2, PRKACA, PTEN) (Figure 3) were found to have a distinct pattern of expression in poly I:C treated organoids resembling the expression profile in BA samples (*p* < 0.05) [10,11,12,13,14,15,16,17,18,19,20,21,22,23,24,25,26,27].

## 4. Discussion

Even though KPE successfully convert BA from a fatal disease to a potentially salvageable condition, its prognosis remains unsatisfactory. It has been recognized that surgery alone could not be the sole treatment for BA. Adjuvant treatment is required to enhance the treatment outcome. An effective treatment for BA has been precluded by a limited understanding of its pathogenesis. Until now, the reported long-term transplant-free survival for this congenital disorder is around 30% to 50% only. Scientific research on BA largely depends on liver tissues obtained during KPE. Apart from its rarity, these liver samples are often fibrotic or even cirrhotic, and could not be processed further for a disease study. Our previous study has successfully demonstrated that BA organoid models could be developed from biopsied liver tissues [9]. Compared to conventional research models, organoids are mini-organs in 3D cultures that recapitulate certain structures and functions of their primary organs. It is particularly useful for translational research in the study of disease development and therapeutic responses. Furthermore, transcriptomic analysis could be performed on these models. For BA liver, a distinct pattern of immune/proinflammatory genes expression could be observed compared with normal liver [28].

The role of viral infection by the Reoviridae genus in BA has been confirmed in previous studies [29]. With our experience in organoid technology, we attempted to expand BA research model by treating control liver organoids with poly I:C. Poly I:C is a synthetic immunostimulant that is structurally similar to a double-stranded RNA (dsRNA) found in the Reovirus and Rotavirus. Its activation can induce immune-mediated inflammation through a number of signaling pathway [30]. In this study, the first finding was the appearance of aberrant morphology observed in BA organoid (27.6% multi-vacuole and 48.3% un-expanded) could be induced by treating control samples with poly I:C at a concentration of 40 ug/mL (15 ± 0.7% multi-vacuole and 66.4 ± 3.5% un-expanded). These morphological changes are unique to BA and are not found in other disorders. To explore the molecular similarity between these two models, we also performed transcriptomic analysis. From the RNA-seq data, we noticed poly I:C liver organoids shared 19 inflammatory genes with BA samples. Further analysis of these overlapping genes confirmed a few showing a high degree of compatibility between the two models. All these genes are responsible for immune-mediated inflammation (Table 2). Combining these findings, we believe that the early inflammatory events in BA can be re-produced from non-BA liver tissues ‘in the dish’. Although poly I:C treated liver organoids could not fully replicate the entire disease process in BA, they still provide an alternative platform for research purposes. Apart from enhancing our understanding of biliary inflammation, the pool of samples can be expanded for pilot study as non-BA liver can also be utilized. Liver samples from genuine BA patients, which are highly precious research materials, could be saved for the later phase of the study when preliminary data are available. In addition, as these samples are originated from a comparatively normal liver which are neither fibrotic nor cirrhotic, there is a higher chance of successful culturing.

We acknowledge there are several limitations in our study. First, being a pilot study, this experiment needs to be repeated on more samples for validation with more mechanistic data. For instance, the effect and interaction with other inflammatory cytokine and Toll-like receptors will need further experiments to investigate. Second, although the control samples were collected from infants, they were not entirely age-matched with the BA samples. Third, poly I:C treated liver organoids could not demonstrate the same disease phenotype and, therefore, are not perfect models to study the disease pathogenesis. Nevertheless, we have demonstrated that these models exhibit morphology and genetic signatures similar to BA liver. They are potential research models for pilot or early phase study to evaluate the inflammatory process while the more precious BA samples could be saved for the later phase of the experiment.

## 5. Conclusions

In summary, we demonstrated that poly I:C (40 µg/mL) treated liver organoids exhibits morphology and genetic signature highly similar to BA organoids. To our best knowledge, this is the first experiment that successfully create a BA-like liver organoid model from non-BA liver. This finding represents a major advancement BA research and this model can potentially expand research materials for BA.

## Figures and Tables

**Table 1:** The diagnosis and liver function of patients whose liver samples were collected.

Diagnosis	Gender	Age at Sample Collection	Total Bilirubin (umol/L)	AST (U/L)	ALT (U/L)	GGT(U/L)
Choledochal cyst	F	8 months	18	42	29	41
Choledochal cyst	F	6 months	10	38	20	30
Hepatoblastoma	F	20 months	9	52	32	18
Hepatoblastoma	M	8 months	12	47	30	22
Biliary atresia	F	63 days	121	68	100	398
Biliary atresia	F	61 days	98	121	112	285

**Table 2:** The list of genes with a high degree of compatibility between BA and poly I:C treated liver organoids and their related actions.

Gene	Action/Related Pathway	Associated Disorders
RELA (RELA Proto-Oncogene, NF-KB Subunit)	Adaptive immunity and responses to pathogens via NF-κB activation.	Inflammatory bowel diseases
SOCS6, SOCS6.1 (Suppressor Of Cytokine Signaling 6)	Inducible by cytokine stimulation, negative regulators of cytokine signaling in a classical negative feedback loop.	Chronic myelogenous leukemia (CML) and erythroleukemia
ARAF (A-Raf Proto-Oncogene, Serine/Threonine Kinase)	Dendritic cells activation and function.	Langerhans cell histiocytosis
CAMK2G (calcium/calmodulin dependent protein kinase II gamma)	Immature T cell lifespan and T cell memory formation.	T cell lymphoma
GNA12 (G Protein Subunit Alpha 12)	Activation of cAMP-Dependent PKA	Familial hyperaldosteronism, Inflammatory bowel diseases
ITGA2 (Integrin Subunit Alpha 2)	Encodes human platelet antigens (HPA) on the surface of platelets and are immunogenic structures.	Neonatal alloimmune thrombocytopenia, Bleeding Disorder, Platelet-Type, 9.
PRKACA (Protein Kinase CAMP-Activated Catalytic Subunit Alpha)	Encodes catalytic protein kinase isoform	Adrenocortical tumor
PTEN (Phosphatase and tensin homolog)	T cells development	Autoimmune disease and lymphoma
